# Integrated Molecular Analysis Indicates Undetectable Change in DNA Damage in Mice after Continuous Irradiation at ~ 400-fold Natural Background Radiation

**DOI:** 10.1289/ehp.1104294

**Published:** 2012-04-26

**Authors:** Werner Olipitz, Dominika Wiktor-Brown, Joe Shuga, Bo Pang, Jose McFaline, Pallavi Lonkar, Aline Thomas, James T Mutamba, Joel S Greenberger, Leona D Samson, Peter C Dedon, Jacquelyn C Yanch, Bevin P Engelward

**Affiliations:** 1 Department of Biological Engineering, Massachusetts Institute of Technology, Cambridge, Massachusetts, USA; 2 Department of Radiation Oncology, University of Pittsburgh, Pittsburgh, Pennsylvania, USA; 3 Department of Nuclear Science and Engineering, Massachusetts Institute of Technology, Cambridge, Massachusetts, USA

**Keywords:** DNA damage, gene expression, *in vivo*, ionizing radiation, low dose-rate, micronucleus assay, mouse

## Abstract

Background: In the event of a nuclear accident, people are exposed to elevated levels of continuous low dose-rate radiation. Nevertheless, most of the literature describes the biological effects of acute radiation.

Objectives: DNA damage and mutations are well established for their carcinogenic effects. We assessed several key markers of DNA damage and DNA damage responses in mice exposed to low dose-rate radiation to reveal potential genotoxic effects associated with low dose-rate radiation.

Methods: We studied low dose-rate radiation using a variable low dose-rate irradiator consisting of flood phantoms filled with ^125^Iodine-containing buffer. Mice were exposed to 0.0002 cGy/min (~ 400-fold background radiation) continuously over 5 weeks. We assessed base lesions, micronuclei, homologous recombination (HR; using fluorescent yellow direct repeat mice), and transcript levels for several radiation-sensitive genes.

Results: We did not observe any changes in the levels of the DNA nucleobase damage products hypoxanthine, 8-oxo-7,8-dihydroguanine, 1,*N*^6^-ethenoadenine, or 3,*N*^4^-ethenocytosine above background levels under low dose-rate conditions. The micronucleus assay revealed no evidence that low dose-rate radiation induced DNA fragmentation, and there was no evidence of double strand break–induced HR. Furthermore, low dose-rate radiation did not induce *Cdkn1a*, *Gadd45a*, *Mdm2*, *Atm*, or *Dbd2*. Importantly, the same total dose, when delivered acutely, induced micronuclei and transcriptional responses.

Conclusions: These results demonstrate in an *in vivo* animal model that lowering the dose-rate suppresses the potentially deleterious impact of radiation and calls attention to the need for a deeper understanding of the biological impact of low dose-rate radiation.

Life has evolved in the midst of a continuous background radiation dose-rate that varies depending on local geological formation and can be further affected by nuclear reactor accidents and nuclear weapons detonations (Hall et al. 2009). As our environment is therefore naturally radioactive, the question becomes: How much additional radiation is too much?

Epidemiological research on low dose-rate radiation has been made difficult by the fact that the biological consequences are subtle and are sometimes obfuscated by inter-individual variation (Mobbs et al. 2011). To overcome this problem, inbred animals housed in controlled conditions have been used to study low dose-rate radiation. Key animal studies show that low dose-rate radiation leads to an increase in the number of antiinflammatory CD4^+^ and CD8^+^ T cells and to an increase in transcription of *Sod1*, the antioxidant gene superoxide dismutase (Ina and Sakai 2005; Tsuruga et al. 2007). Moreover, fractionated low dose radiation over a period of several weeks has been shown to increase the number of T-regulatory cells (Tago et al. 2008; Tsukimoto et al. 2008). Radiation-induced up-regulation of antiinflammatory immune cells has been associated with a lower frequency of lymphomas (Courtade et al. 2002; Ina et al. 2005; Lacoste-Collin et al. 2007; Mitchel 2007; Nakatsukasa et al. 2008; Tago et al. 2008; Tsukimoto et al. 2008; Tsuruga et al. 2007). In contrast, however, a higher frequency of hematological malignancies and chromosome aberrations has been reported in mice and dogs after continuous low dose-rate irradiation (Seed et al. 2002a, 2002b; Tanaka et al. 2007, 2008, 2009). Thus, it remains unclear to what extent (and at what dose-rate) low dose-rate radiation affects cancer risk.

Of particular interest is radiation-induced DNA damage. Carcinogenic radiation exposures are known to induce DNA strand breaks and chromosomal rearrangements (Bekker-Jensen and Mailand 2010; Chadwick and Leenhouts 2011; Holland et al. 2011). Importantly, a single acute dose of radiation can give rise to cancer over a decade later, which is consistent with DNA damage being predictive of downstream cancer risk (Ron 1998). Therefore in this study, we have focused on measurements of DNA damage and DNA damage responses.

Here we show that despite continuous exposure of mice to radiation at a dose that is approximately 200-fold higher than the permissible exposure limit determined by the International Commission on Radiological Protection (ICRP 2007) there was no significant change in the levels of DNA base lesions, homologous recombination (HR), micronucleus frequency, or transcriptional stress responses. Our findings suggest that exposure to continuous radiation at a dose-rate that is orders of magnitude higher than background does not significantly affect several key measures of DNA damage and DNA damage responses.

## Materials and Methods

*Radiation exposure of mice.* Three- and 7-week-old C57BL6 mice were purchased from Taconic Farms, Inc. (Hudson, NY) and acclimatized for 1–2 weeks before experiments. Fluorescent yellow direct repeat (FYDR) mice and positive-control FYDR-Rec mice in the C57BL6 background were bred in-house. All animals were housed in pathogen-free barrier facilities and treated humanely with regard for alleviation of suffering. Experimental cohorts included a 1:1 male-to-female ratio, and litters were split into treatment and control groups. Group sizes for base lesion analysis, gene expression analysis, and micronucleus assay were 6, 16, and 6 animals, respectively. Group sizes for the HR assay were 60 and 24 animals for the continuous radiation and acute exposure experiments, respectively. Two treatment conditions were used throughout the experiments: *a*) continuous low dose-rate radiation, and *b*) acute radiation exposure. For low dose-rate exposures, 4-week-old animals were exposed for 5 weeks using an Iodine-125 (^125^I)-based variable low dose-rate irradiator (Olipitz et al. 2010). Briefly, to create a large, uniform exposure area, commercially available plexan boxes (flood phantoms; Biodex Medical Systems Inc., Shirley, NY) were filled with ^125^I in a sodium hydroxide (NaOH) buffer. Flood phantoms were placed below the animal cages, resulting in a dose-rate of 0.00017 cGy/min ± 0.00002 [see Supplemental Material, Figure S1 (http://dx.doi.org/10.1289/ehp.1104294)]. For acute exposures, 9-week-old mice were irradiated for 1.4 min at a dose-rate of 7.1 cGy/min using a Philips RT250 X-ray machine (Philips Medical Systems, Bothell, WA) at 75 kV with a 0.2-mm Cu filter in place. All exposed mice received a total dose of 10.5 cGy.

*DNA base lesion analysis.* All animals were sacrificed by carbon dioxide (CO_2_) euthanasia immediately after cessation of radiation exposure. Their spleens were removed, and splenic DNA was isolated using a DNA isolation Kit for Cells and Tissues (Roche Diagnostic Corporation, Indiana, IL). All buffers were supplemented with the deaminase inhibitors coformycin (5 µg/mL) (National Cancer Institute, Bethesda, MD) and tetrahydrouridine (50 µg/mL) (Calbiochem, San Diego, CA), and the antioxidant desferrioxamine (0.1 mM) (Sigma-Aldrich Corp., St. Louis, MO) (Pang et al. 2007). 8-Oxo-7,8-dihydro-2´-deoxyguanosine (8-oxodG), 2´-deoxyinosine (dI), 1,*N*^6^-etheno-2´-deoxyadenosine (εdA), and 3,*N*^4^-etheno-2´-deoxycytidine (εdC) were analyzed using liquid chromatography–coupled tandem mass spectrometry (LC-MS/MS) as previously described (Pang et al. 2007). Briefly, DNA was enzymatically hydrolyzed to 2´-deoxynucleosides that were resolved by reversed-phase LC, with fractions containing the 2´-deoxynucleosides collected at empirically determined elution times. Individual 2´-deoxynucleosides in the LC fractions were then analyzed by isotope-dilution tandem quadrupole mass spectrometry using internal standards and calibration curves based on defined molecular transitions.

*Gene expression analysis.* Blood samples were drawn from individual 4-week-old mice before continuous low dose-rate radiation exposure by retroorbital bleeding and immediately after cessation of radiation exposure by terminal heart puncture. For acute exposure experiments, retroorbital bleeding was performed on 8-week-old animals, which were subsequently exposed at 9 weeks of age and sacrificed immediately after radiation exposure. White blood cells (WBCs) were isolated as previously described (Olipitz et al. 2002) except that whole mouse blood was lysed twice in lysis buffer (Sigma-Aldrich Corp.) for 6 min on ice. WBCs were washed in phosphate buffered saline (PBS), resuspended in 100 µl RNAlater (Qiagen, Hilden, Germany) and stored at –80°C. RNA was isolated using a commercially available kit (RNeasy; Qiagen). cDNA was generated using an archive kit (High Capacity cDNA RT Kit; Applied Biosystems, Foster City, CA). Using *Gapdh* (glyceraldehyde-3-phosphate dehydrogenase) as an internal control, relative gene expression was assessed using the Taqman system on an AB7100 thermal cycler (Applied Biosystems). For low dose-rate studies, there were 16 animals per group. For acute irradiations, two experiments were performed, each with 6 animals per group.

*Bone marrow micronucleus assay* in vivo. Mice were humanely euthanized by CO_2_ asphyxiation immediately after cessation of continuous low dose-rate radiation and 24 hr after acute radiation exposure, and the bone marrow was removed from the femurs and tibiae. A single-cell suspension was generated by mechanical dissociation, passed through a cellulose column, spread onto a slide, fixed in 25^o^C methanol for 10 min, and stained with acridine orange (Fisher Scientific, Hanover Park, IL) at a concentration of 20 µg/mL in 19 mM sodium phosphate (NaH_2_PO_4_) and 81 mM sodium phosphate, dibasic (Na_2_HPO_4_), for 10 min at 4^o^C. Slides were washed for 10 min in 4^o^C staining buffer, air dried, stored at 4^o^C, and subsequently examined using a Labophot microscope (Nikon, Garden City, NY). Representative micrographs were acquired using a Sony DSC-P93A Cyber-Shot digital camera (Sony Group, Minato, Tokyo, Japan). Acridine orange–stained cells were scored using a 40× oil-immersion objective and fluorescence (100 W mercury lamp excitation). The cytologist was blinded to the identity of the slides and differential cell counting was used to enumerate relevant cell types and thus quantify the percentage of micronucleated polychromatic erythrocytes (MN-PCEs) among total PCEs. PCEs, also known as reticulocytes, still contain RNA and thus fluoresce red after acridine orange stain, allowing them to be distinguished from mature red blood cells (RBCs; faint green) and nucleated cells (bright yellow). MN-PCE contain small amounts of nuclear DNA that is left behind when an erythroid progenitor undergoes DNA damage while differentiating into a PCE. More than 2,000 PCEs were scored per slide and experiments were performed in duplicate, each with 6 animals per group.

*Analysis of HR frequency in pancreatic tissue.* FYDR mice carry a direct repeat recombination substrate that contains two differently mutated copies of the coding sequence for enhanced yellow fluorescent protein (Eyfp) (Hendricks et al. 2003). An HR event can restore the full length *Eyfp* coding sequence, yielding a fluorescent cell. The positive-control FYDR-Rec mice arose spontaneously through a recombination event in a gamete and all cells within the positive-control mice carry the full length *Eyfp* cDNA. The frequency of fluorescent yellow recombinant cells can be assessed using flow cytometry analysis of disaggregated pancreatic tissue, or by *in situ* imaging (Wiktor-Brown et al. 2006a). Briefly, pancreata were harvested immediately after cessation of continuous low dose-rate exposure and 3.5 weeks after acute radiation exposure. The period of 3.5 weeks was designed for potential radiation-induced HR events to occur and to adjust for previously determined age-related increases in HR events (both continuously exposed animals and acutely exposed animals were of the same age at analysis). Pancreata were compressed to a uniform thickness of 0.5 mm and images were taken under a 1× objective on a Nikon 600 eclipse fluorescent microscope. Using Adobe Photoshop 5.0 (Adobe Systems, San Jose, CA) images were then adjusted for brightness and contrast and compiled to represent the entire area of a pancreas. Fluorescent spots were then counted in a blinded fashion. For flow cytometry analysis, pancreata were dissociated into a single-cell suspension and analyzed on a BD FACScan flow cytometer (Becton, Dickinson and Co., Franklin Lakes, NJ) as previously described (Wiktor-Brown et al. 2006a). Statistical analysis was performed using Student’s *t*-test or Mann–Whitney *U* test, as appropriate.

## Results

*Variable low-dose irradiator.* A recently developed ^125^I-based low dose-rate irradiator provides an effective method to continuously expose mice to low dose-rate radiation (Olipitz et al. 2010). While ^125^I is not a radionuclide found in nature, its photon emissions are a reasonable surrogate for both background radiation [the majority of background radiation tracks through our bodies are photon tracks] and environmental contamination [the radionuclide of most concern for long-term contamination after nuclear reactor accidents or nuclear weapons explosions is cesium-137 (^137^Cs), a photon emitter].

We previously showed that the average dose-rate delivered to the animals across the phantom is 0.00017 cGy/min ± 0.00002 (Olipitz et al. 2010). This dose-rate is approximately 400 times higher than background radiation and approximately 200 times higher than the ICRP’s 1-year limit for radiation workers (ICRP 2007). However, it is still considered to be a low dose-rate as it is only about 5 times the level of natural radiation found in certain places, such as Iran (Ghiassi-Nejad et al. 2002), and it is also lower than the dose-rate known to affect cancer and longevity in animals studies (National Council on Radiation Protection and Measurements 1980). An exposure period of 5 weeks was chosen to reach a cumulative dose of 10.5 cGy, because approximately 10 cGy of ionizing radiation delivered acutely has been shown to affect DNA damage endpoints (Abramsson-Zetterberg et al. 1996; Amundson et al. 2000; Bhilwade et al. 2004; Gruel et al. 2008; Uma Devi and Sharma 1990).

*DNA base-lesion levels in splenic tissue.* Radiation-induced reactive oxygen species (ROS), such as hydroxyl radical (OH^•^), superoxide radical (O_2_^•–^), and hydrogen peroxide (H_2_O_2_), can create mutagenic and cytotoxic DNA base lesions (Halliwell and Aruoma 1991). In addition, the cellular damage caused by ionizing radiation can potentially cause inflammation, with local generation of high levels of reactive nitrogen species (RNS), including nitric oxide (NO), nitrous anhydride (N_2_O_3_), and peroxynitrite (ONOO^–^) (Dedon and Tannenbaum 2004). While ONOO^–^ causes DNA oxidation, N_2_O_3_ can cause nitrosative deamination of DNA nucleobases (Dedon and Tannenbaum 2004). We therefore set out to determine the extent to which continuous low dose-rate radiation affects DNA damage levels by direct or indirect mechanisms that potentially modulate the formation or clearance of DNA damage.

**Figure 1:**
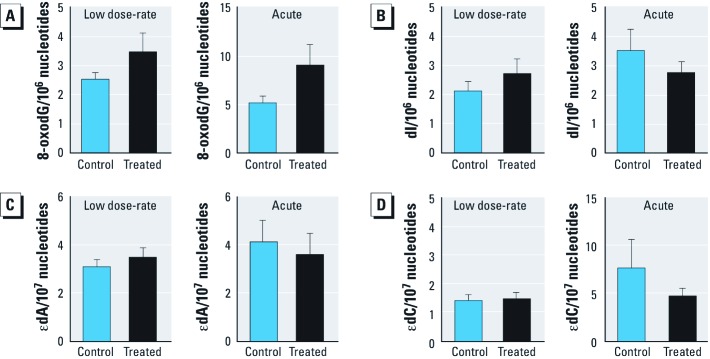
Exposure to 10.5 Gy acute (7.1 cGy/min) and chronic irradiation (0.0002 cGy/min) does not change steady-state base lesion levels. Effects of chronic, low dose-rate and acute irradiation on DNA base lesion levels of (*A*) 8-oxodG, (*B*) dI, (*C*) εdA, and (*D*) εdC were measured by LC-MS/MS in splenic DNA. Data represent mean ± SE for *n *= 6 and were analyzed by Student’s *t*-test.

LC-MS/MS is highly sensitive and can be used to measure the steady-state levels of DNA lesions (Dedon et al. 2007). In the present study, we quantified mutagenic and cytotoxic base lesions, including 8-oxodG (a DNA oxidation product), dI (a nucleobase deamination product), and εdA and εdC (two lesions derived from reactions of DNA with lipid peroxidation products). The spleen was chosen for analysis given its radiosensitivity. After exposure to approximately 400-fold background radiation for 5 weeks, we did not detect any significant changes in the levels of base lesions in spleen tissue from irradiated mice (Figure 1).

One possible reason that base damage might not accumulate is that radiation-induced DNA damage may be rapidly repaired. We therefore asked if the same total dose of radiation induces base damage when delivered acutely, at a dose-rate that was approximataely four orders-of-magnitude higher (7.1 cGy/min). Even under acute conditions, we did not detect any significant difference in the levels of base lesions (Figure 1). Together these results show that exposure to 10.5 cGy does not significantly affect the levels of several key DNA base lesions that are known to be formed in response to radiation and inflammation, regardless of the dose-rate (ranging from 0.0002 to 7.1 cGy/min).

**Figure 2:**
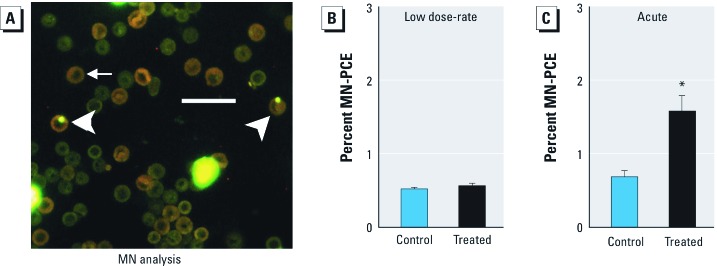
Representative image of a PCE-containing micronuclei (MN-PCE; arrowheads) and of a normal RBC (arrow) isolated from bone marrow; bar = 20 µm (*A*). Low dose-rate (0.0002 cGy/min) irradiation (*B*) does not induce micronuclei in PCEs, whereas acute (7.1 cGy/min) irradiation (*C*) does. Data (mean ± SE ) are representative of two independent experiments; percent MN-PCE was calculated from > 2,000 scored PCE per sample. *p < 0.05 using unpaired two-tailed Student’s *t*-test.

*Micronuclei analysis in RBCs.* Although far less frequent than radiation-induced base lesions, radiation-induced double strand breaks (DSBs) are severely cytotoxic and mutagenic (Helleday et al. 2007). The micronucleus assay is an exquisitely sensitive approach for detecting DSBs (Hayashi et al. 2000). Using the *in vivo* RBC micronucleus assay, small chromosomal fragments can be detected in enucleated RBCs (Figure 2A) (Kirsch-Volders et al. 2000). To explore the impact of dose-rate on susceptibility to DSBs, we compared the extent to which 10.5 cGy radiation induces micronuclei when delivered acutely versus chronically. Consistent with previous studies, exposure to 10.5 cGy delivered acutely (7.1 cGy/min) resulted in a significant increase in micronuclei in mice *in vivo* (*p* < 0.005) (Figure 2C) (Abramsson-Zetterberg et al. 1996; Bhilwade et al. 2004; Uma Devi and Sharma 1990). In contrast, no significant increase in micronuclei was observed in continuously irradiated mice (Figure 2B). These data reveal that dose-rate can significantly affect radiation-induced DNA damage levels.

**Figure 3:**
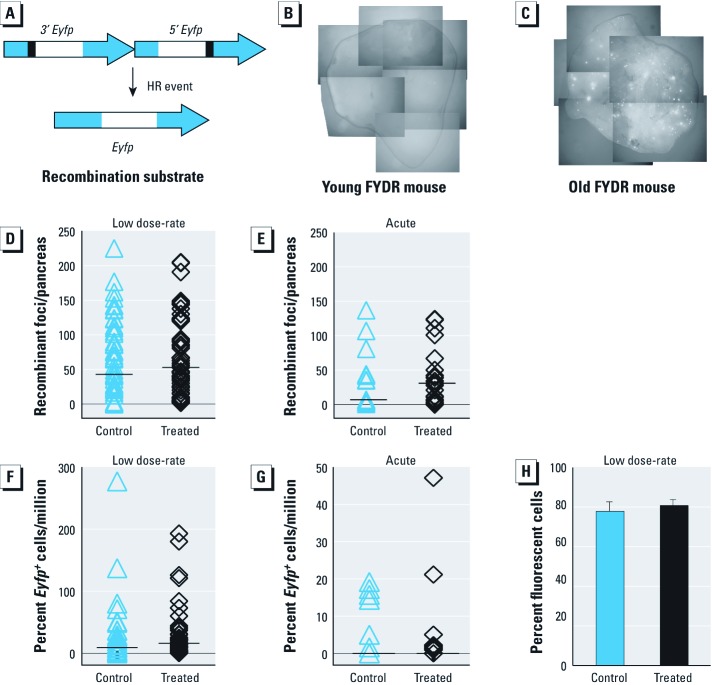
FYDR mice carry a recombination substrate (*A*) that results in expression of *Eyfp* upon recombination repair. The *Eyfp* signal can be detected by *in situ* imaging. The frequency of *Eyfp*^+^ cells increases with age [(*B*) 4-week-old (young) mouse; (*C*) 24-week-old (old) mouse]. Chronic, low dose-rate (0.0002 cGy/min; *D,F*) and acute (7.1 cGy/min; *E,G*) irradiation do not affect HR frequency in the pancreas; ^+^, positive. Chronic irradiation does not affect *Eyfp* expression in FYDR control mouse (*H*). Values are mean ± SE; statistical analysis was performed using two-tailed Mann–Whitney *U* test.

*Frequency of HR events in the pancreas.* An alternative approach for studying DSBs is to assess DSB repair activity. We have recently developed FYDR mice that allow investigation of mitotic HR, one of the major DSB repair pathways in mammals (Wiktor-Brown et al. 2006a, 2006b). FYDR mice carry a direct repeat recombination substrate for which an HR event can restore the full length *Eyfp* coding sequence (Figure 3A) (Hendricks et al. 2003). The frequency of fluorescent yellow recombinant cells can be assessed using *in situ* imaging or flow cytometry (Figure 3A–C). Recombinant cells can continue to fluoresce for their lifespan, making it possible to monitor the accumulation of recombinant cells over time (Wiktor-Brown et al. 2006b). Thus, although induction of recombination can potentially be detected by an increase in the frequency of recombinant cell foci (compare Figure 3B,C), no difference was observed in the frequency of HR among low dose-rate irradiated and non-irradiated animals (Figure 3D,F).

Although these data suggest that low dose-rate radiation did not affect the frequency of HR, it remained formally possible that radiation caused silencing of the *Eyfp* gene (Suzuki et al. 2011), which could lead to a false negative result. We therefore exploited FYDR-Rec positive-control mice to test for radiosuppression of *Eyfp* expression; however, no suppression was detected (Figure 3H). Therefore, we conclude that low dose-rate radiation does not significantly affect HR.

To explore the possibility that acute exposure might induce HR, animals were exposed to 10.5 cGy at a dose-rate of 7.1 cGy/min. Although there appears to be a slight increase in HR frequency by *in situ* imaging, the difference is not statistically significant (Figure 3E,G). Taken together, our analysis of DSB repair indicates that long-term low dose-rate irradiation at approximately 400-fold background for 5 weeks does not lead to a detectable increase in the frequency of either micronuclei or HR.

**Figure 4:**
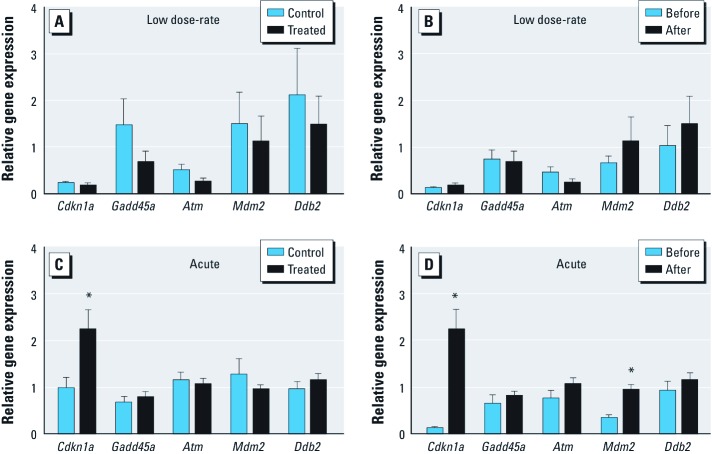
Effects of chronic, low dose-rate (0.0002 cGy/min; *A,B*) and acute (7.1 cGy/min; *C,D*) ionizing radiation on gene expression in WBCs. Gene expression changes were compared between control and treated groups after irradiation (*A,C*) and in irradiated animals before and after irradiation (*B,D*). Data (mean ± SE) are representative of two independent experiments. *p < 0.05 using unpaired two-tailed Student’s *t*-test (*A*,*C*) and paired two-tailed Student’s *t*-test (*B*,*D*).

*Gene expression analysis of DNA damage response genes.* Gene expression changes have been observed in response to acute irradiation delivered at doses as low as 1 cGy (Alvarez et al. 2006; Amundson et al. 2000, 2001; Fujimori et al. 2005). Several genes found to be consistently affected by radiation are part of the transformation related protein 53 [*p53* (*Trp53*)] DNA damage response: cyclin-dependent kinase inhibitor 1A (*Cdkn1a*), growth arrest and DNA-damage-inducible 45 alpha (*Gadd45a*), transformed mouse 3T3 cell double minute 2 (*Mdm2*), ataxia telangiectasia mutated homolog (human) (*Atm*), and damage specific DNA binding protein 2 (*Ddb2*) (Gruel et al. 2008). As WBCs are particularly responsive to radiation exposure (Amundson et al. 2000, 2003), we assessed gene expression levels for *Cdkn1a*, *Gadd45a*, *Mdm2*, *Atm*, and *Ddb2* in primary WBCs after exposure to low dose-rate radiation (0.0002 cGy/min). We found that there was no significant difference in gene expression between irradiated and non-irradiated animals for any of the five genes (Figure 4A). To explore the impact of dose-rate, we exposed mice to 10.5 cGy irradiation delivered acutely (7.1 cGy/min). At this higher dose-rate, *Cdkn1a* was significantly up-regulated (Figure 4C), indicating that DNA damage responses are dose-rate dependent, which is consistent with previous studies (Amundson et al. 2003).

A significant challenge for all animal studies is variability due to interindividual differences. We therefore developed an approach for a paired analysis, wherein blood samples were collected from the same animals both before and after radiation exposure. Regardless of whether the data was paired or pooled, *Cdkn1a* was significantly induced by acute irradiation, though we detected a greater induction using the paired experimental design (compare Figure 4C,D). Furthermore, using paired analysis conditions, we also detected a significant increase in expression of *Mdm2* (Figure 4D). These studies suggest that longitudinal assessment increases the sensitivity of the assay to subtle changes in gene expression. Nevertheless, under the conditions of low dose-rate exposure (0.0002 cGy/min), there were no significant changes in gene expression, even with a paired analysis (Figure 4B).

Taken together, studies of animals that live under conditions of prolonged continuous exposure to radiation at approximately 400-fold background do not show any evidence of increased levels of base damage (for 8-*oxodG*, *dI*, ε*dA*, ε*dC*) nor DSBs (micronuclei and HR), nor induction of a DNA damage response (at the level of *p53*-inducible gene expression). Importantly, when delivered acutely, the same total dose induced micronuclei and induced key genes involved in the DNA damage response.

## Discussion

In the event of radioactive contamination, the majority of the population will be exposed to low dose radiation over extended periods of time (United Nations Scientific Committee on the Effects of Atomic Radiation 2000). Despite appreciation of the importance of preparedness, the biological effects of continuous low dose radiation are poorly understood [for excellent reviews on the biological impact of low dose radiation, see Mobbs et al. (2011); Muirhead et al. (2009); Vrijheid et al. (2007); Wall et al. (2006)]. In the present study we have explored the impact of continuous low dose-rate radiation through studies of DNA damage and responses in an animal model.

Based on published studies, we estimate that the steady-state level of base lesions is approximately 10,000/cell, whereas exposure to 10.5 cGy is only expected to induce approximately 400 base lesions/cell (Pouget et al. 1999, 2002). LC-MS/MS is an exquisitely sensitive method to detect DNA base lesions and has been successfully used to detect base-lesion levels after exposure to ionizing radiation and other ROS/RNS generating conditions such as chronic inflammation (Frelon et al. 2000; Pang et al. 2007; Pouget et al. 2002). Although directly induced lesions may be too low to be detectable above background, it remained possible that radiation could indirectly alter the steady-state levels of damage by changing the physiological state of the tissue or by modulating DNA repair. However, steady-state base-lesion levels in splenic DNA were not changed as compared to non-irradiated controls. Additionally, the same total dose given at a high dose-rate (7.1 cGy/min) did not affect base-lesion levels. Taken together, this is the first time that base lesions have been measured *in vivo* after low dose-rate radiation, and there was no significant impact on the steady-state levels of several key DNA base lesions.

DSBs are highly cytotoxic and mutagenic and potentially result in deletions, chromosomal translocations or loss of heterozygosity that can promote cancer (Friedberg et al. 2006; Goodhead 1994; Helleday et al. 2007; Ward 1988). The micronucleus assay is a sensitive assay that detects chromosome breaks (Hayashi et al. 2000). Consistent with published studies (Abramsson-Zetterberg et al. 1996; Bhilwade et al. 2004; Uma Devi and Sharma 1990), we observed radiation-induced micronuclei in acutely exposed animals (10.5 cGy at 7.1 cGy/min). However, when the same total dose was delivered continuously at a very low dose-rate of 0.0002 cGy/min, no significant differences in micronuclei frequency were observed between the irradiated and control cohort. Micronuclei persist for 24 hr after exposure, after which time the mature RBCs enter the blood stream, cycling for approximately 120 days. Thus, under chronic exposure conditions one would not only detect micronuclei induced by the most recent radiation exposure, but also those micronuclei in RBCs that re-enter the highly perfused bone marrow. Thus, even though the micronucleus assay is highly radiation sensitive and has the potential to detect accumulated DNA damage, low dose-rate radiation did not induce micronuclei in this study.

As an alternative approach for analysis of DSBs, we assayed for induction of HR by low dose-rate radiation. We found that when 10.5 cGy was delivered either at a low dose-rate or acutely, it did not induce HR in the pancreas. Assuming a linear relationship between the number of DSBs and the total dose, a radiation dose of 10 cGy will induce about two DSBs per cell (Hall 2000), which is likely below the limits of detection. Nevertheless, the FYDR mouse studies can also be used to detect changes in steady-state levels of HR, which could be affected by exposure (e.g., by induction of an adaptive response). Thus, low dose-rate radiation neither directly nor indirectly induced HR in this study.

Acutely delivered low dose radiation has been shown to induce transcriptional changes at doses as low as 1 cGy (Amundson et al. 2000, Fujimori et al. 2005; Gruel et al. 2008). The most sensitive and most consistently radiation-affected genes belong to the DNA damage response network (Alvarez et al. 2006; Amundson et al. 2000, 2003; Gruel et al. 2008). In an attempt to address the consequences of a protracted radiation exposure to low doses, Besplug and coworkers exposed mice to a daily acute dose of 5 cGy to simulate chronic, low dose-rate exposure. Importantly, after 10 days of irradiation the strongest transcriptional response was found in genes of the p53 signaling network, similar to acute exposure effects (Besplug et al. 2005). We therefore used a group of genes known to be induced by low dose radiation (*Cdkn1a*, *Gadd45a*, *Mdm2*, *Ddb2*, and *Atm*), to query gene expression changes in WBCs. Interestingly, we did not detect a significant difference in gene expression between irradiated and control groups. This result indicates that exposure to approximately 400-fold background radiation is not sufficient to affect radiation-sensitive genes in DNA damage response pathways, a finding consistent with the absence of a stress response.

To increase the sensitivity of our approach for detecting radiation-induced changes in gene expression, we used a paired analysis approach that suppresses inter-individual differences. Although two genes were found to be induced under acute conditions, there was no change in gene expression under low dose-rate conditions. Such a dose-rate threshold has been described previously in studies of the hematopoietic system of dogs. Below a threshold dose-rate of 0.0002 cGy/min (approximately the same as the dose-rate used in the present study) dogs did not display any changes in bone marrow morphology, whereas dogs exposed to dose-rates above this threshold displayed severe hematopoietic dysfunction, e.g., aplastic anemia, myeloproliferative disease, leukemia (Seed et al. 1981, 2002a, 2002b). Taken together, continuous low dose-rate radiation not only shows a dose-rate threshold for cell morphology (Seed et al. 2002a, 2002b) but also for DNA damage responses.

Despite the use of highly sensitive assays for DNA damage responses, it remains possible that genetic changes are induced by low dose-rate radiation, but that such changes are below the limits of detection for the assays used. Chromosome aberrations offer an alternative approach for detecting chromosome breaks, and using this approach, others have shown that low dose-rate radiation indeed induces aberrations *in vitro* (although the dose-rate was approximately 10-fold higher than that used in the present study) (Tanaka et al. 2009). In addition, it is also important to consider the possibility that the biological impact of DNA damage varies according to the type of radiation. While most DSBs are rapidly repaired, a minor proportion of breaks are associated with additional DNA lesions. Such complex breaks have been shown to be resistant to DNA repair (Asaithamby et al. 2011; Sutherland et al. 2000) and thus may persist at undetectable levels. High linear energy transfer (LET) radiation induces more complex breaks compared to low LET radiation (such as that used in this study) (Hall 2000), although elevated radiation levels from a contaminated environment result primarily in additional exposure to low-LET radiation (particularly from ^131^I and ^137^Cs). Nevertheless, the current study has important limitations in terms of the types of assays selected and the focus upon specifically low LET radiation. These limitations must be taken into consideration with regard to the potential of radiation exposure on human health.

Exposure to radiation is inevitable. In the present study, we assessed the effect of long-term low dose-rate radiation on genomic stability using several highly sensitive end points for DNA damage and DNA damage responses. Using some of the most sensitive techniques available, low dose-rate radiation (approximately 400-fold natural background radiation) over a 5-week period does not affect DNA base-lesion levels, micronuclei formation, HR frequency, or expression of DNA damage response genes. Importantly, an equal dose of radiation delivered acutely did induce DNA damage and DNA damage responses, thus demonstrating in an *in vivo* animal model that lowering the dose-rate suppresses the potentially deleterious impact of radiation. Current U.S. policy dictates that a dose-rate of approximately 30-fold higher than background is too high to be permissible for human habitation (Federal Emergency Management Agency 2008). Given the enormous costs associated with making constraints on public policy too stringent (or too loose), these studies point to a significant need for additional knowledge regarding the impact of low dose-rate radiation.

## Supplemental Material

(332 KB) PDF
