# Supplementary material for: Integrated Molecular Analysis Indicates Undetectable Change in DNA Damage in Mice after Continuous Irradiation at ~ 400-fold Natural Background Radiation
Source: Environ Health Perspect. 2012 Apr 26;120(8):1130–6. doi: 10.1289/ehp.1104294 (PMC3440074; doi:10.1289/ehp.1104294)

Supplemental Material

Integrated Molecular Analysis Indicates Undetectable DNA Damage in Mice after Continuous Irradiation at ~400-fold Natural Background Radiation

Werner Olipitz, Dominika Wiktor-Brown, Joe Shuga, Bo Pang, Jose McFaline, Pallavi Lonkar, Aline Thomas, James T. Mutamba, Joel S. Greenberger, Leona D. Samson, Peter C. Dedon, Jacquelyn C. Yanch, Bevin P. Engelward

Figure S1

The variable low dose-rate irradiator consists of a plastic cart (PC), holding an aluminum tray (T) and flood phantom (P). The phantom is filled with radioactive liquid and serves as the radiation source. A steel cart (SC) holding the cages fits exactly above the plastic cart. A leaded acrylic sheet (A) mounted on the steel cart ensures radiation protection for experimenters and animal husbandry staff when handling the steel cart. Details on handling of the low dose-rate irradiator, use of ^{125}I as the radioactive source, dose-rate calculations, dosimetry and uniformity of radiation exposure can be found elsewhere (Olipitz et al. 2010).

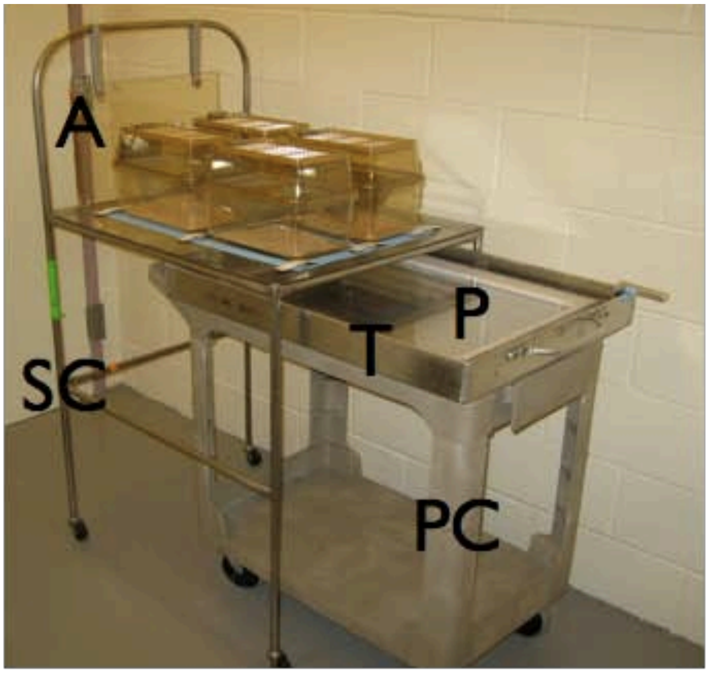

Supplement: (332 KB) PDF [file ehp.1104294.s001.pdf]
